# Continuous intrathecal saline infusion for treating refractory spontaneous intracranial hypotension: A case report

**DOI:** 10.37796/2211-8039.1417

**Published:** 2023-12-01

**Authors:** Po-Fan Chiu, Yu-Hsiang Lin, Hui-Shan Lu, I-Han Hsiao, Hung-Lin Lin

**Affiliations:** Department of Neurosurgery, China Medical University Hospital, Taichung, Taiwan

**Keywords:** Cerebrospinal fluid, Continuous intrathecal saline infusion, Epidural blood patch, Orthostatic headache, Spontaneous intracranial hypotension

## Abstract

Spontaneous intracranial hypotension (SIH) is a poorly understood condition that presents with a wide variety of symptoms, ranging from mild headaches to coma. It is typically caused by continuous spontaneous leakage of spinal cerebrospinal fluid (CSF), resulting in orthostatic headaches. However, the appropriate management of refractory SIH remains unclear. A 50-year-old man presented with orthostatic headache followed by a rapid decline in mental status. The imaging findings were consistent with the diagnosis of SIH, with bilateral cerebral subdural hematomas and abnormal fluid collection in the posterior epidural space from the T2 to T12 levels.

Computed tomography myelography of the whole spine revealed multiple high-flow CSF leakages at the T6 to T8 levels. Despite treatment with bilateral burr hole drainage for subdural hematomas and repeated lumbar epidural blood patch (EBP) three times, the patient’s condition worsened and he developed stupor. A lumbar intrathecal saline bolus (90 ml) was administered to restore CSF depletion. The patient’s verbal function improved immediately, and continuous intrathecal saline infusion was administered at a rate of 10 ml/h for two days. The patient’s stupor gradually resolved, and after his symptoms improved, the EBP injection was repeated at the T8 level. The patient recovered completely, and during the six-year follow-up, there were no signs of recurrence. SIH may cause a refractory decline in mental status, and lumbar intrathecal saline infusion may help arrest or reverse an impending central (transtentorial) herniation. This case demonstrates an appropriate bolus and continuous infusion of normal saline, and documents the resolution of SIH. This maneuver may change the CSF flow pattern and aims to seal the CSF fistula. Further studies are needed to better understand the mechanism of intrathecal saline infusion and establish effective treatment strategies for refractory cases of SIH.

## 1. Introduction

Spontaneous intracranial hypotension (SIH) is a rare disorder first described by Georges Schaltenbrand in 1938 [1,2]. Symptoms and signs range widely from mild headaches to altered mental status [3–5]. Spontaneous cerebrospinal fluid (CSF) leakage is considered the most common cause [3,5]. The diagnostic criteria were based on the International Classification of Headache Disorders, 3rd edition, and included spontaneous headache associated with CSF leakage (brain/spine imaging) or CSF pressure <60 mmH_2_O [6].

Treatment options include conservative management (complete bed rest and hydration), spine epidural blood patches, and direct surgical repair [3,5,7]. However, the appropriate management of obtundation caused by SIH is not well defined, and the treatment of refractory SIH can be challenging. In this case report, we present our experience with continuous intrathecal saline infusion for the treatment of refractory SIH that caused obtundation.

## 2. Case report

**Fig. 1:**
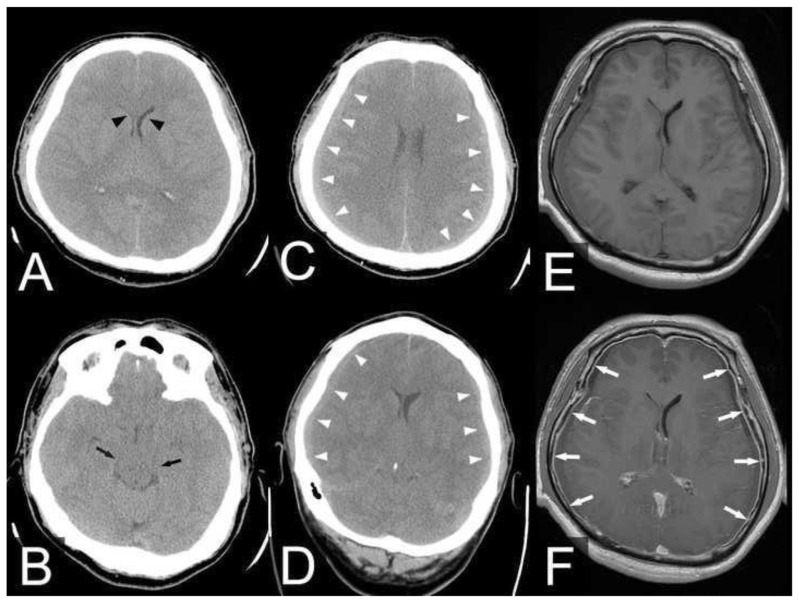
(A, B) Brain CT on Day 1 of hospitalization showed a slit ventricle (black arrowhead) and saggy brain with basal cistern obliteration (black arrow). (C, D) Brain CT on Day 3 of hospitalization showed bilateral subdural fluid collection (white arrowhead) with mass effect. (E) Non-contrast brain MRI T1-weighted image showed bilateral subdural fluid collection. (F) Contrast brain MRI T1-weighted image showed pachymeningeal enhancement (white arrow).

A 50-year-old man presented with sudden onset of severe headache, with a visual analog scale (VAS) score of 8 out of 10, for three days. The headache was located in the left occipital and frontal areas and was not accompanied by nausea, vomiting, tenderness, weakness, numbness, visual defects, or hearing loss. The headache was orthostatic, it improved when lying flat and exacerbated when sitting. Neurological examination results were all within normal limits. Brain computed tomography (CT) revealed a slit ventricle and a crowded suprasellar cistern (Fig. 1). The patient was admitted to the hospital for suspected SIH.

**Fig. 2:**
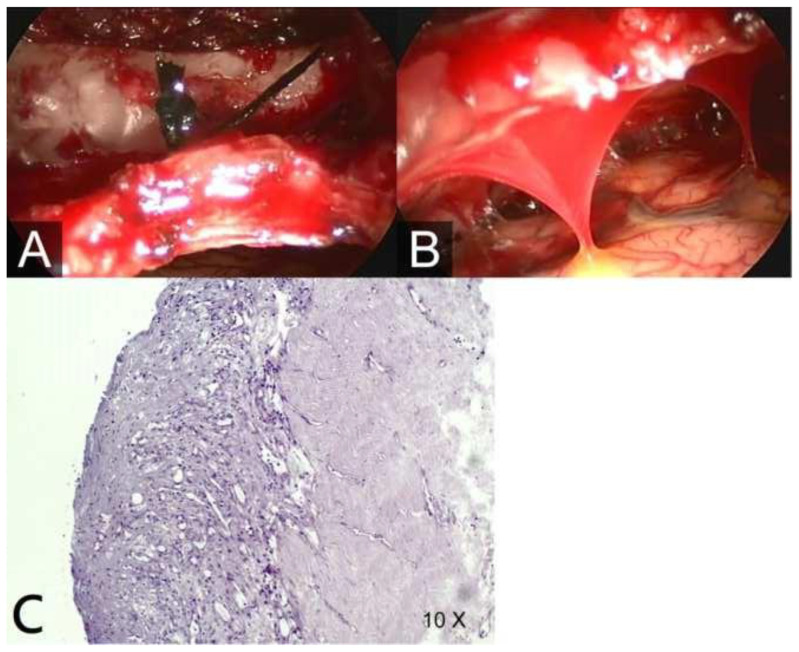
(A) Remarkable thickened dura under intraoperative endoscopic view with (B) some connection between the dura and brain surface. (C) Fibrosis and focal fibrinoid exudate admixed with mild lymphocytes in the dura mater.

After admission, the patient’s headache improved with multiple analgesics and bed rest, with the head of the bed at a 30-degree angle. On Day 4 of hospitalization, the patient’s mental status deteriorated to drowsiness, with a Glasgow Coma Scale (GCS) score of 7 (E1V1M5). Brain CT and magnetic resonance imaging (MRI) scans were performed, which showed bilateral subdural hematoma on the CT scan and diffuse pachymeningeal enhancement with subdural fluid collection on the MRI scan (Fig. 1). Bilateral craniotomy with subdural drainage was performed. A large amount of mixed yellowish and dark red subdural fluid was evacuated under increased pressure and sluggishness of the brain surface was noted. During the dural opening, a thickened dura mater was noted (Fig. 2), and a leptomeningeal biopsy was performed. Microscopic examination revealed marked fibrosis and focal fibrinoid exudate mixed with mild lymphocytes (Fig. 2). A 7 mm Jackson–Pratt (JP) drain was inserted as a closed suction drain system to evacuate the residual subdural hematoma, and it was removed 5 days later due to minimal drainage. An intracranial pressure (ICP) monitor was placed in the right hemispheric parenchyma, which showed low ICP (−5~5 mmHg) postoperatively.

**Fig. 3:**
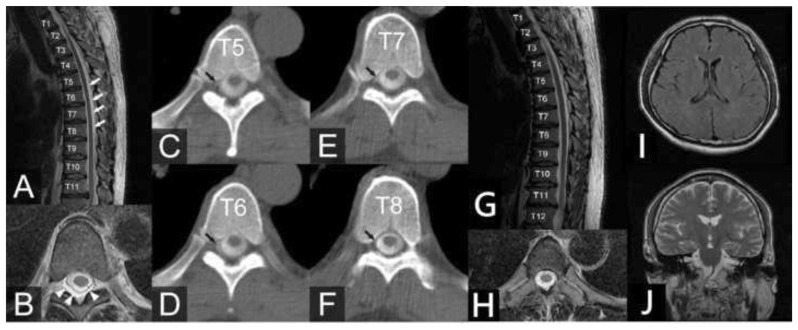
(A) MR sagittal image shows the dura (white arrows) located between the CSF at the T1 to T12 levels. (B) MR axial view shows epidural CSF collection (white arrow heads). (C, D, E, F) Thoracic CT myelography shows contrast leakage at the T5 to T8 levels on the right side (black arrows). (G) The thoracic spine sagittal T2weighted image 6 months after treatment shows no more epidural fluid collection. (H) The thoracic spine MRI axial image 6 months after treatment shows no more epidural fluid collection. (I, J) The brain MRI image 6 months after treatment shows no more subdural fluid collection.

After surgery, the patient’s mental status improved, and his GCS score returned to 15 (E4V5M6). Thoracic spine MRI showed abnormal fluid collection in the posterior epidural space from the T2 to T12 levels (Fig. 3). CT myelography demonstrated focal abnormal contrast collection in the right lateral aspect of the dura sac at the T5 to T8 levels, and multiple high-flow CSF leakages were suspected (Fig. 3). Therefore, an autologous epidural blood patch (EBP) with 20 ml was injected into the T8 level on Day 9 of hospitalization. However, on Day 15 of hospitalization, the patient complained of headaches again, and his mental status deteriorated, with a GCS score of 13 (E3V4M6). Brain CT showed bilateral recurrence of the subdural hematoma with a mass effect. Consequently, the patient underwent a second bilateral craniotomy with subdural drainage. A 7 mm JP drain was reinserted to evacuate the subdural hematoma, and it was removed 5 days after the operation. The postoperative ICP was approximately −5~5 mmHg.

After the second surgery, EBP was performed by injecting autologous blood with 20 ml twice at the T8 level on Days 17 and 23 of hospitalization. However, despite EBP injections, the patient’s ICP remained low (5~5 mmHg) and the GCS score was 10 (E3V1M6), with no obvious improvement in mental status. His mental status further deteriorated to a GCS score of 7 (E1V1M5) on Day 26 of hospitalization. Therefore, a lumbar catheter was inserted at the L3 to L4 levels, and a 90 ml intrathecal saline bolus was infused the next day to bring the ICP from −5 mmHg to the normal range (around 10–15 mmHg). His GCS score improved from E1V1M5 to E4V4M6 immediately after infusion. A continuous intrathecal saline infusion was started at a rate of 10 ml/h to keep his ICP around 10–15 mmHg. Two days later, his mental status became clear, with a GCS score of 15 (E4V5M6), and he had no more headaches. A fourth EBP was injected at the T8 level with 20 ml of autologous blood. The intrathecal saline infusion was discontinued two days later, and no further deterioration in mental status or headache was noted. The patient was discharged on Day 37 of hospitalization without any neurological deficits. Six months later, brain CT and thoracic spine MRI (Fig. 3) were normal, and no SIH recurrence was observed at the six-year follow-up.

## 3. Discussion

SIH is an uncommon disease and there are no established guidelines for its treatment. Conservative treatments, such as bed rest, hydration, and caffeine, have a success rate of approximately 28% [3]. When conservative treatments fail, EBP is the treatment of choice for SIH [3,5,7]. The success rate of the first EBP injection is approximately 64%, with non-targeted and targeted EBP showing similar outcomes [3]. If the first EBP fails, a repeat EBP can be attempted after 5–7 days [8–10]. Surgical repair is less common and is reserved for patients in whom the CSF leakage site can be localized and those in whom EBP failed [3,5,7].

However, managing obtundation caused by SIH and treating refractory SIH are challenging. There are few studies on intrathecal saline infusion for treating refractory SIH, but it has been reported to be effective in temporarily reversing obtunded mental status [4,11–16]. Another study reported that combining continuous intrathecal saline infusion and repeating targeted and non-targeted EBP resulted in a dramatic but temporary clinical improvement [17].

In our case, we repeated EBP three times for the patient, but his condition did not improve and his GCS score decreased. The continuous intrathecal saline infusion was used to treat his obtundation mental status, which returned dramatically. We then combined this with EBP, and the patient recovered without recurrence during the six-year follow-up. A common hypothesis is that successful SIH treatment with EBP results from inflammation in the regional dura that seals small leaks, but large recurrent or refractory spontaneous intracranial hypotension probably requires intrathecal saline infusion and large volume EBP to seal large leaky dural defects.

**Fig. 4:**
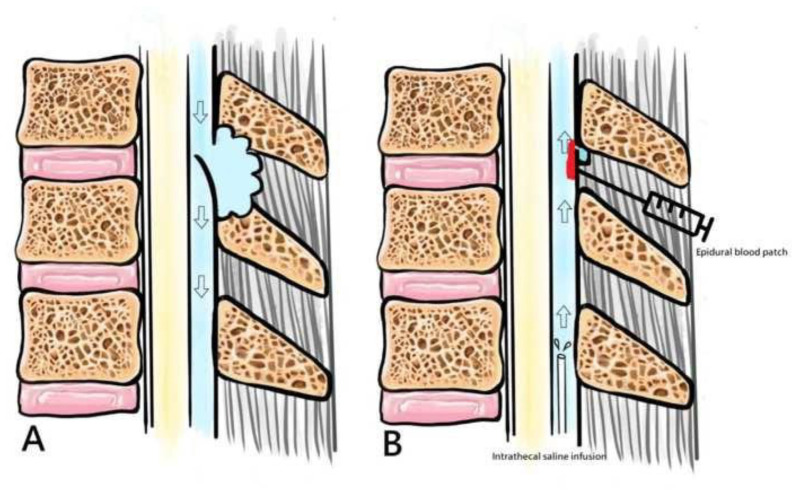
A schematic diagram for the CSF flow; (A) posterior wall retracts due to relatively low ICP during the diastolic phase, resulting in the one-way valve opening; (B) Successful intrathecal saline infusion may be result in changing the net CSF flow and pressure to refill the intrathecal space, thereby re-positioning leaky dural flap to reduce any defect and improve the possible success of subsequent EBP.

The direction of net CSF flow in the spinal canal has been debated in the literature for decades [18]. Recent studies suggest that CSF flow oscillates back and forth, but the net CSF flow in the spinal canal is directed caudad [19–21]. Many articles suggest that some CSF is absorbed at the spinal level, so the net CSF flow is craniocaudal [18,20,22]. Therefore, we hypothesized that, in the setting of spinal CSF leakage, the CSF flows out of the spinal canal and the CSF flow is craniocaudal (Fig. 4). Successful intrathecal saline infusion may be due to changes in the CSF flow, which pushes the dural flap back and reduces the dural defect (Fig. 4), thereby making EBP more effective in sealing the defect.

In this case, we share our experience of successfully treating SIH with continuous intrathecal saline infusion combined with EBP. However, further studies are needed to better understand the mechanism of intrathecal saline infusion and establish effective treatment strategies for refractory cases of SIH.

In conclusion, SIH can lead to a refractory decline in mental status, and lumbar intrathecal saline infusion may be beneficial for halting or reversing impending central (transtentorial) herniation. This case illustrates the successful use of an appropriate bolus and continuous infusion of normal saline, which resulted in the resolution of SIH. The intrathecal saline infusion may alter CSF flow patterns and aid in sealing CSF fistulas. Further research is necessary to better understand the mechanisms underlying intrathecal saline infusion and to establish effective treatment strategies for refractory SIH.

## Data Availability

Data sharing not applicable to this article as no datasets were generated or analysed during the current study.
